# Transcriptome Dynamics of Double Recessive Mutant, *o2o2o16o16*, Reveals the Transcriptional Mechanisms in the Increase of Its Lysine and Tryptophan Content in Maize

**DOI:** 10.3390/genes10040316

**Published:** 2019-04-23

**Authors:** Wei Wang, Yi Dai, Mingchun Wang, Wenpeng Yang, Degang Zhao

**Affiliations:** 1 The State Key Laboratory Breeding Base of Green Pesticide and Agricultural Bioengineering, The Key Laboratory of Plant Resources Conservation and Germplasm Innovation in Mountainous Region (Ministry of Education), Guizhou University, Guiyang 550025, China; wwmaize@126.com (W.W.); daiyi96@126.com (Y.D.); 2 Guizhou Institute of Upland Food Crops, Guiyang Station for DUS Testing Center of New Plant Varieties (MOA), Guizhou Academy of Agricultural Sciences, Guiyang 550006, China; wangmingchun64@163.com

**Keywords:** *opaque2*, *opaque16*, lysine, tryptophan, RNA-Seq, *Zea mays* L.

## Abstract

In maize, pyramiding of *o2* and *o16* alleles can greatly improve the nutritional quality of grains. To dissect its molecular mechanism, we created a double recessive mutant line, *o2o2o16o16*, by introgression of the *o2* and *o16* alleles into the wild-type maize inbred line, by molecular marker-assisted backcross selection. The kernels (18 day after pollination (DAP), 28 DAP, and 38 DAP) of the *o2o2o16o16* mutant and its parent lines were subject to RNA sequencing (RNA-Seq). The RNA-Seq analysis revealed that 59 differentially expressed genes (DEGs) were involved in lysine metabolism and 43 DEGs were involved in tryptophan metabolism. Among them, the genes encoding AK, ASADH, and Dap-F in the lysine synthesis pathway were upregulated at different stages of endosperm development, promoting the synthesis of lysine. Meanwhile, the genes encoding LKR/SDH and L-PO in the lysine degradation pathway were downregulated, inhibiting the degradation of lysine. Moreover, the genes encoding TAA and YUC in the tryptophan metabolic pathway were downregulated, restraining the degradation of tryptophan. Thus, pyramiding *o2* and *o16* alleles could increase the lysine and tryptophan content in maize. These above results would help to uncover the molecular mechanisms involved in the increase in lysine and the tryptophan content, through the introgression of *o2* and *o16* alleles into the wild-type maize.

## 1. Introduction

Corn (*Zea may* L.) is the main feed and food crop, worldwide, with its quality directly affecting the healthy development and edibility by human beings, livestock, and poultry. The nutritional quality of corn grains generally refers to the nutritional components in corn grain, including protein, fat, starch, and various vitamins, as well as mineral element contents. Among them, lysine, from proteins, is a necessary amino acid for humans and monogastric animals. The content of lysine in wild-type maize is low (less than 0.3%) and cannot meet the nutritional quality requirements for human consumption and food processing (more than 0.5%). Furthermore, it cannot even meet the requirements for the content of lysine in livestock and poultry feed (0.6–0.8%) [1].

Singleton and Jones first discovered the mutation of *opaque1* (*o1*) and *opaque2* (*o2*) in maize. Mertz et al. found that the mutation of *o2* could significantly increase the content of lysine in the endosperm of maize seeds [2]. The *O2* gene, located on chromosome 7, encodes the transcription factors of the basic leucine zipper family, recognizes the promoter of the zein gene through the *O2* box (TCCACGT) and regulates the expression of 22 kDa α-zein and 15 kDa β-zein genes [3,4]. Following this, new similar mutations were discovered successively, such as *floury1* (*fl1*) [5], *floury2* (*fl2*) [6], and *floury3* (*fl3*) [7], *opaque4* (*o4*) [8,9], *opaque5* (*o5*) [10,11], *opaque6* (*o6*) [12,13], *opaque7* (*o7*) [14,15,16], *opaque15* (*o15*) [17], *proline1* (*pro1*) [18], De*-B30 [19], and *Mc* [19], amongst others. However, it is difficult to use these mutations in breeding and production, due to the death of seedlings or complex genetic patterns. To expand upon the available high-lysine corn germplasm resources, we previously applied *Mu* transposon technology to create a new high-lysine mutation, *opaque16* (*o16*), which has a lysine content of 0.36%. This gene is located on the long arm of chromosome 8, between molecular markers umc1141 and umc1121 [20].

To further improve the nutritional quality of maize, the *o16* and *o2* genes were pyramided by a marker-assisted selection (MAS) using Qi205 (*o2o2*) and QCL3021 (*o16o16*) as parents. The content of lysine in the grain increased by 6% and 30%, compared with that of the *o2* parent and *o16* parent, respectively [20]. Zhang et al. used Tai19 (*o2o2*) as the recurrent parent and QCL3021 (*o16o16*) as the donor parent. Marker-assisted backcross selection (MABS) were then used to introgress the *o16* gene into the *o2* lines, which allowed us to obtain 17 families, with increases in lysine content of 22.33% and 65.86%, compared with Tai 19 (*o2o2*) and QCL3021 (*o16o16*), respectively [21]. Sarika et al. used MABS to introgress *o2* and *o16* genes into different recurrent parents. Compared with the recurrent parents, the content of lysine and tryptophan increased by 76% and 91%, respectively [22,23]. Therefore, the polymerization of high-lysine mutant genes could improve the nutritional quality of corn seeds.

The introgression of *o2* gene into wild-type maize with different genetic backgrounds could cause varying degrees of changes in transcription patterns [24]. The *o2* gene introgression into waxy corn could not only cause varying degrees of physiological, biochemical, and proteomic changes, but could also change the expression of endosperm proteins, amino acids, biosynthesis, starch, stress response, and signal transduction [25]. The *O2* gene-regulated multiple metabolic pathways related to biological process and molecular function during waxy maize endosperm development—*Zm00001d029385.1* and *Zm00001d012262.1*—which encode the EF-1α and LHT1, were upregulated, but the gene that encodes sulfur-rich proteins was downregulated in the *o2o2wxwx* lines, raising the waxy grain lysine content [26]. In addition, the pyramiding of the *o2* and *o7* genes could affect amino acid metabolism; carbon metabolism; storage protein synthesis, transcription and translation; and signal transduction [27]. However, it is unknown which physiological and metabolic changes are induced by the pyramiding of *o2* and *o16* genes, and what the underlying molecular mechanisms are that result in the increase in lysine.

To dissect the molecular mechanisms behind the improved quality of the maize double recessive mutant line (*o2o2o16o16*), the *o2* and *o16* genes were introgressed into wild-type maize lines by MABS. The kernels of the *o2o2o16o16* line and their parents (18 DAP, 28 DAP, and 38 DAP) were subject to RNA sequencing (RNA-Seq). The transcriptional differences between the double recessive mutants (*o2o2o16o16*) and the wild-type parent were compared. The results provided a foundation for studying the underlying molecular mechanisms that underlay the improvement in grain nutrient quality through the introgression of the *o2* and *o16* genes.

## 2. Materials and Methods

### 2.1. Plant Materials

CML530 is a maize inbred line (wild-type, WT) from CIMMYT. QCL8003_2 is a double mutant line (*o2o2o16o16*) bred by our research group. Using CML530 as a recurrent parent and QCL8003_2 as a donor parent, the *o2o2o16o16* mutant was converted to the CML530 background, through two backcrossing cycles, using MABS. Co-dominant SSR markers phi112 and umc1141, were used to select heterozygous (*O2o2O16o16*) individuals at BC_1_F_1_ and BC_2_F_1_, while selecting homozygous (*o2o2o16o16*) individuals at BC_2_F_2_, which was followed by several rounds of self-pollination. The bred *o2o2o16o16* mutant line was named QCL8011_2. According to the 55 K SNP microarray analysis, the recovery rate of the genomic genetic background of QCL8011_2 (*o2o2o16o16*) was 92.62% to QCL530, which was higher than the theoretical background recovery rate (87.50%). 

The maize inbred line CML530 and the endosperm mutant line QCL8011_2 (*o2o2o16o16*) in the CML530 background, were grown in an experimental field in the Guizhou Academy of Agricultural Sciences (Guiyang, China) (N26°30′14″ and E106°39′21″) during spring 2017. 

Generally, kernels are sampled at 18 DAP for RNA-Seq of maize endosperm, such as that done by Zhou et al. [25]. In order to dissect the transcriptome dynamics of *o2o2o16o16* mutant at different stages of endosperm development, the kernels of QCL8011_2 (*o2o2o16o16*) and CML530 (WT) were again sampled at 28 DAP and 38 DAP. A minimum of five well-filled kernels for each genotype were sampled at 18 DAP, 28 DAP, and 38 DAP, and then immediately frozen in liquid nitrogen for RNA extraction.

Mature kernels were harvested after physiological maturity and dried in a drying room. To minimize the effect of biological variation between ears, equal numbers of four well-filled ears were pooled and treated as one sample for nutritional evaluation.

### 2.2. Kernel Characteristics and Submicroscopic Structure

The mature and dry kernels of QCL8011_2 and CML530 were selected to observe the ear and kernel under natural light, while the transparency of the grain was observed on the light box. The kernel was cross cut by a blade and the farinaceous and keratinous degree of kernel was observed under natural light and photographed.

After the peel was removed from the mature and dry kernels, a blade was used to cut the endosperm from the middle and a small piece of endosperm was taken. An ion sputtering instrument (E-1010, Hitach, Tokyo, Japan) was used to embed platinum, which was subsequently observed under scanning electron microscopy (SEM) (S3400N, Hitach, Tokyo, Japan) in the Guizhou Key Laboratory of Agricultural Biotechnology.

### 2.3. Amino Acid Analyses

Amino acid contents of mature maize grains were analyzed using an automatic amino acid analyzer (S-433D, Sykam, Munich, Germany). Approximately 120–150 mg of the dried powder of each sample was weighed and transferred to a glass tube with a cover (20 mL), before 5 mL of HCL (6 M) was added. Each tube was incubated at 110 °C, in a water bath for 24 h. After this was cooled to room temperature, the pH was adjusted to 2.0 and the solution was diluted to a volume to 100 mL using ddH_2_O. Each sample was then filtered through the membrane (0.45 μm) into the liquid chromatographic sample bottle (2 mL) and used for determination of the contents of 17 free amino acids (FAAs), which were aspartate (Asp), threonine (Thr), serine (Ser), glutamate (Glu), glycine (Gly), alanine (Ala), cysteine (Cys), valine (Val), methionine (Met), isoleucine (Ile), leucine (Leu), tyrosine (Tyr), phenylalanine (Phe), histidine (His), lysine (Lys), arginine (Arg), and proline (Pro).

Tryptophan (Trp) content was measured using a spectrophotometer (Synergy 2, Biotek, Vermont, USA) by reading the absorbance at 590 nm, according to the national standard (GB7650-87 of China). Three replicates were performed for each sample.

### 2.4. RNA-Sequencing Library Construction and Sequencing

The total RNA of the whole kernels from 18 DAP, 28 DAP, and 38 DAP was isolated using a plant RNA kit (OMEGA), according to manufacturer’s protocol. The concentration and quality of each RNA sample was checked using an Agilent 2100 Bioanalyzer (Agilent Technologies, Palo Alto, CA, USA). The first step in the workflow involves purifying the poly(A)-containing mRNA molecules using poly(T) oligo-attached magnetic beads. After purification, the mRNA was fragmented into small pieces, using divalent cations at a high temperature. The cleaved RNA fragments were used in the synthesis of first strand cDNA, using reverse transcriptase and random primers. This was followed by second strand cDNA synthesis using DNA Polymerase I and RNase H. A single ‘A’ base was then added to these cDNA fragments, followed by subsequent ligation of the adapter. The products were then purified and enriched using PCR amplification. We then quantified the PCR yield by Qubit and pooled samples together to make a single strand DNA circle (ssDNA circle), which gave the final library. DNA nanoballs (DNBs) were generated with the ssDNA circle, by rolling circle replication (RCR), to enlarge the fluorescent signals during the sequencing process. The DNBs were loaded into the patterned nanoarrays and they were read using a single-end read of 50 bp on the BGISEQ-500 platform, for the following data analysis study. For this step, the BGISEQ-500 platform combined the DNA nanoball-based nanoarrays and stepwise sequencing, using the combinatorial probe-anchor synthesis sequencing method. Transcriptome sequencing was carried out on a BGISEQ-500 sequencing platform by Shenzhen Huada Gene Technology Co. Two replicates of each sample were sequenced.

### 2.5. Identification of Differentially Expressed Genes

In this study, low-quality reads and reads containing adapters or having an unknown base percentage >10% were removed from the original sequencing data, using SOAPnuke soft (v1.5.2, https://github.com/BGI-flexlab/SOAPnuke) [28]. The resulting clean reads were aligned to the reference genome (B73 version 4) [29] using HISAT (v2.0.4, Hierarchical Indexing for Spliced Alignment of Transcripts) [30] and aligned to the reference gene using Bowtie2 (v2.2.5, http://bowtie-bio.sourceforge.net/Bowtie2/index.shtml) [31], to calculate the gene alignment rate. FPKM (fragments per kilobase million) was calculated as the expression level of genes and transcripts by using RSEM software (v1.2.12, http://deweylab.biostat.wisc.edu/RSEM) [32]. NOISeq2 [33] was used to identify differentially expressed genes (DEGs). DEGs were screened according to the following criteria—fold change ≥ 2 and corrected *p* ≤ 0.05.

### 2.6. Gene Ontology and Pathway Enrichment Analysis of Differentially Expressed Genes

Gene Ontology (GO) enrichment analysis provided all GO terms that were significantly enriched in a list of DEGs compared to a genome background, and filtered the DEGs that corresponded to specific biological functions. This method firstly mapped all DEGs to the GO terms in the database (http://www.geneontology.org/), calculating gene numbers for every term, before using the hypergeometric test to find significantly enriched GO terms in the input list of DEGs. In this study, the calculated *p* values were subjected to the Bonferroni correction [34], and GO terms that were enriched in DEGs were identified, based on *p* ≤ 0.05. Typically, functions with FDR ≤ 0.01 were considered to indicate significant enrichment.

Pathway enrichment analysis of DEGs was performed with the guidance of the Kyoto Encyclopedia of Genes and Genomes (KEGG) database (https://www.genome.jp/kegg/). KEGG pathway enrichment was calculated in the same way as the GO functional enrichment analysis [35]. In this study, pathways with a *p* value ≤ 0.05 and FDR ≤ 0.01 were considered to be significantly enriched in DEGs.

### 2.7. qRT-PCR Analysis

A total of 28 DEGs were selected for qRT-PCR verification at 18 DAP, 28 DAP, and 38 DAP. The primers, shown in Table S1, were designed online (https://sg.idtdna.com/site/account/login?returnurl=%2Fprimerquest%2FHome%2FIndex). Total RNA used for RNA-Seq was reversely transcribed into cDNA, using a Thermo Scientific RevertAid First Stand cDNA Synthesis (BIO-RAD, Hercules, CA, USA). Quantitative analysis was conducted using CFX Connect Real-Time PCR System (BIO-RAD, Hercules, CA, USA), according to the method used by Liu et al. [36]. Three replicates were used for each sample and actin was used as an internal standard. The 2^−ΔΔCT^ method was used to calculate relative changes in gene expression.

### 2.8. Accession Numbers

The raw data of RNA-Seq reads were deposited in the National Center for Biotechnology Information (NCBI) database, under the accession number PRJNA 517535. Biosample accessions were as follows: SAMN10836467, SAMN10836468, SAMN10836469, SAMN10836470, SAMN10836471, SAMN10836472, SAMN10836473, SAMN10836474, SAMN10836475, SAMN10836476, SAMN10836477, and SAMN10836478. 

## 3. Results

### 3.1. Kernel Characteristics and Submicroscopic Structure

**Figure 1:**
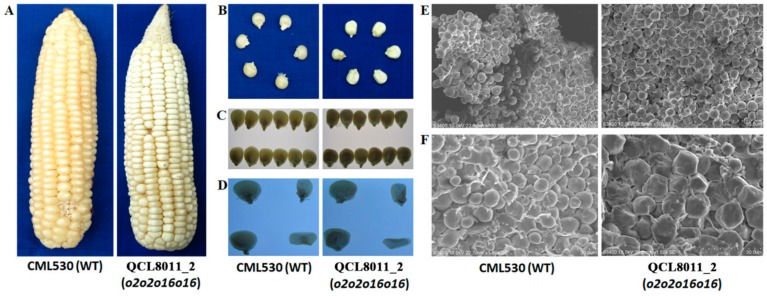
Phenotypic features of CML530 and QCL8011_2. (**A**) Photographs of intact ears taken under normal light. (**B**) Photographs of mature kernels taken under normal light. (**C**) Light transmission of mature kernels on a light box. (**D**) Cross-sections of mature kernels on a light box. (**E**) Scanning electron micrograph for endosperms of QCL8011_2 and CML530 at 700× magnification. (**F**) Scanning electron micrograph for endosperms of QCL8011_2 and CML530 at 1500× magnification.

The ear characteristics of the *o2o2o16o16* mutant line, QCL8011_2, and its recurrent parent CML530 were observed under natural light, while the phenotypes and cross-sections of the kernels were observed under projected light. The ear of QCL8011_2 was a cylindrical type (Figure 1A), the kernel was dull and basically opaque (Figure 1B,C) and the endosperm was farinaceous (Figure 1D). The kernel of CML530 showed a smooth and shiny seed coat, a full keratinous endosperm, and a vitreous seed (Figure 1B–D).

Scanning electron microscopy (SEM) showed that the starch granules of QCL8011_2 were mostly irregular in shape and slightly larger in size than that of CML530. There was a high density of matrix proteins that were scattered with the starch granules in the gap. The endosperm starch granules of CML530 were mostly ellipsoidal or spheroidal, with a low density of matrix proteins that encapsulated starch granules (Figure 1E,F).

### 3.2. Changes of Free Amino Acids Composition in o2o2o16o16 Endosperm

**Figure 2:**
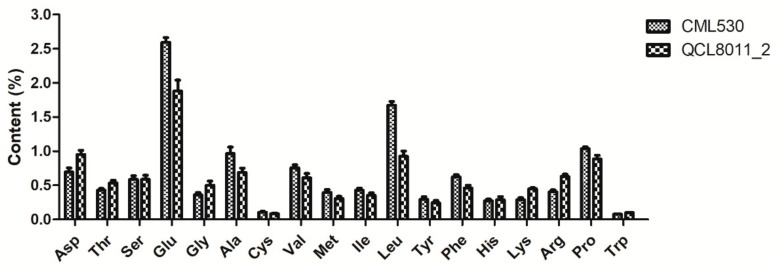
Contents of 18 free amino acids (FAAs) in mature kernels of CML530 (*O2O2O16O16*) and QCL8011_2 (*o2o2o16o16*).

Compared with CML530, the contents of all 17 FAAs changed to different degrees in the QCL8011_2 mutant line. Figure 2 shows that Asp, Thr, Ser, Gly, His, Lys, and Arg contents were higher in QCL8011_2. Among them, Lys, Arg, Gly, Asp, and Trp greatly increased by 56.08%, 55.16%, 38.75%, 36.96%, and 22.17%, respectively. However, Leu, Ala, Glu, Phe, Met, Val, Cys, Ile, Tyr, and Pro contents were decreased. Among them, Leu, Ala, Glu, Phe, and Met greatly decreased by 44.65%, 28.57%, 27.63%, 26.30%, and 22.55%, respectively.

### 3.3. The Quality of RNA-Sequencing 

To dissect the regulation network of the *o2o2o16o16* line, kernels (18 DAP, 28 DAP, and 38 DAP) of the QCL8011_2 and CML530 were used for the RNA-Seq analysis. Two biological replicates were used in this study. For each replicate, we generated 23,984,698 clean reads on average (Table S2). A total of 86.76% clean reads were successfully aligned to the maize B73 reference genome (*Zea mays* L. AGPv4.38) and 81.27% clean reads were distributed in the gene regions (Table S3). The average Q20 and Q30 (the percentage of the number of bases with sequencing base mass value greater than 20 or 30, respectively, out of the total number of bases in the original data) of all samples were 97.58% and 90.17%, respectively (Table S4).

More than 29,000 genes were detected in each sample, which corresponded to more than 70% of the total number of genes (Figure S1 and Table S5). The correlation coefficients among the two sequencing repeats were more than 0.95 (Figure S2), which indicated that the transcriptome sequencing data in all samples were highly reliable and, thus, suitable for analyzing differentially expressed genes.

### 3.4. Identification of Differentially Expressed Genes 

**Figure 3:**
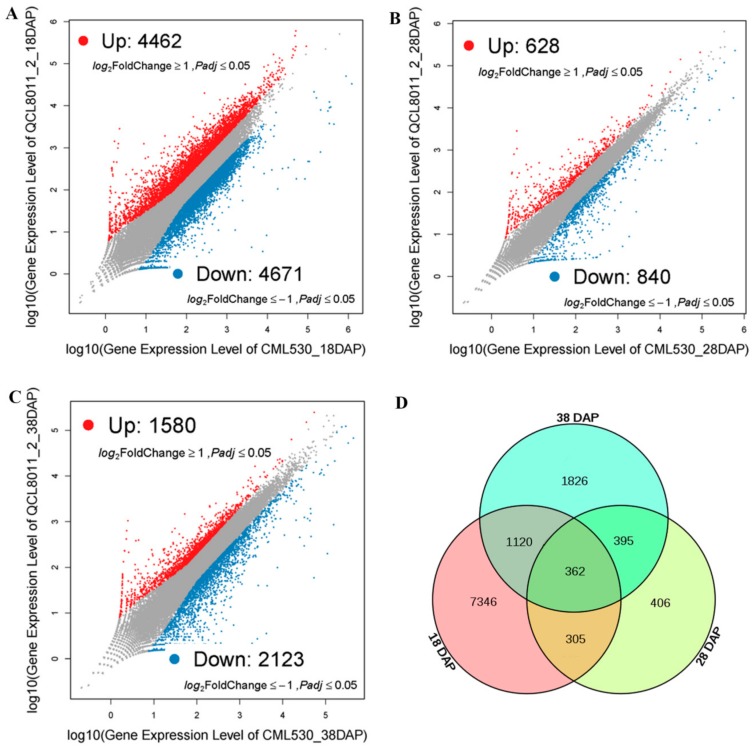
(**A**) Scatter plots of differentially expressed genes (DEGs) in CML530 vs. QCL8011_2 at 18 day after pollination (DAP). (**B**) Scatter plots of DEGs in CML530 vs. QCL8011_2 at 28 DAP. (**C**) Scatter plots of DEGs in CML530 vs. QCL8011_2 at 38 DAP. (**D**) Venn diagram of DEGs in CML530 vs. QCL8011_2 at 18 DAP, 28 DAP, and 38 DAP.

To identify significantly DEGs, we compared the transcript abundance in QCL8011_2 relative to CML530, using the NOISeq software. Compared with CML530, 9,133 genes were differentially expressed in QCL8011_2 at 18 DAP, among which 4,462 were upregulated and 4,671 were downregulated (Figure 3A). A total of 1,468 genes were differentially expressed in QCL8011_2 at 28 DAP, among which 628 were upregulated and 840 were downregulated (Figure 3B). A total of 3,703 genes were differentially expressed in QCL8011_2 at 38 DAP, among which 1,580 were upregulated and 2,123 were downregulated (Figure 3C). A total of 667 DEGs were detected at both 18 DAP and 28 DAP, 757 DEGs were found at both 28 DAP and 38 DAP, 1,482 DEGs at both 18 DAP and 38 DAP, and 362 DEGs were detected at all three 18 DAP, 28 DAP, and 38 DAP (Figure 3D).

### 3.5. Functional Annotations of Differentially Expressed Genes

**Figure 4:**
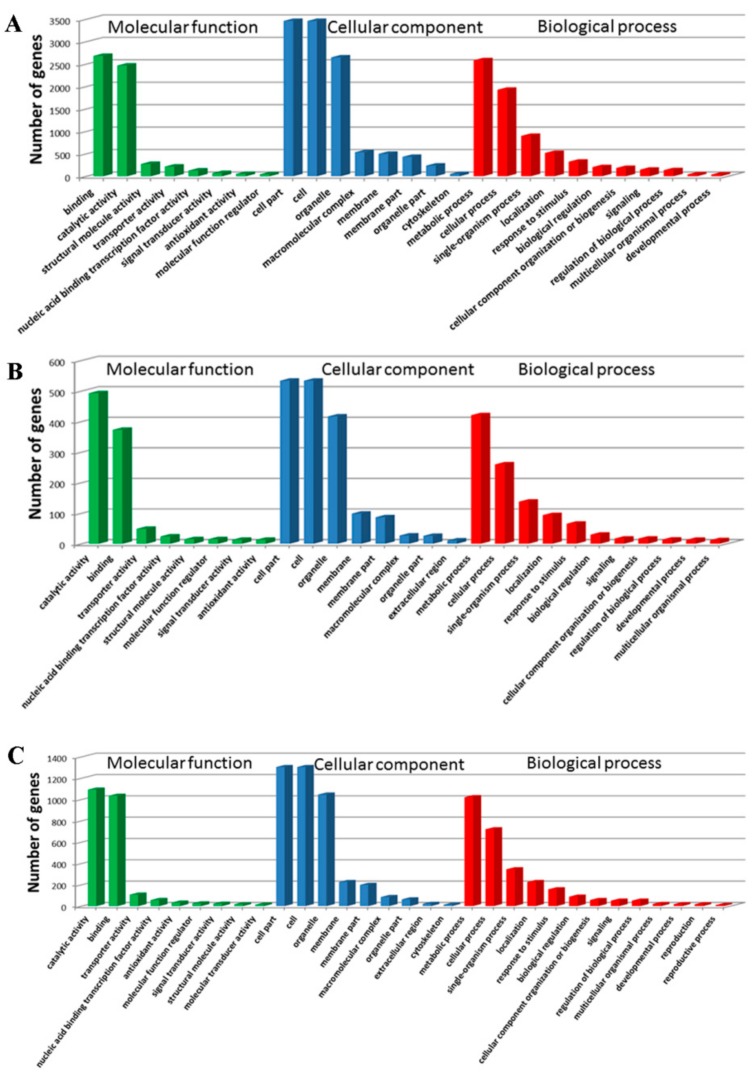
The GO analysis of DEGs in CML530 vs. QCL8011_2 at 18 DAP (**A**), 28 DAP (**B**), and 38 DAP (**C**). X axis represents GO terms. Y axis means number of DEGs. All GO terms are grouped in to three ontologies—brown is for molecular function, blue is for cellular component, and orange is for biological process.

The WEGO software was used to classify the DEGs at 18 DAP, 28 DAP, and 38 DAP into GO functional categories related to the biological process (BP), cellular component (CC), and molecular function (MF). At 18 DAP, a total of 5,639 DEGs were classified into 20 terms (BP), 11 terms (CC), and 11 terms (MF) (Figure 4A). At 28 DAP, a total of 944 DEGs were classified into 18 terms (BP), 11 terms (CC), and 10 terms (MF) (Figure 4B). At 38 DAP, a total of 2,240 DEGs were classified into 18 terms (BP), 11 terms (CC), and 11 terms (MF) (Figure 4C). In BP, the DEGs were mainly enriched in metabolic process (GO: 0008152) and cellular process (GO: 0009987). In CC, the DEGs were mainly enriched in the cell (GO: 0005623), cell part (GO: 0044464), and organelle (GO: 0043226). In MF, the DEGs were mainly enriched in binding (GO: 0005488) and catalytic activity (GO: 0003824).

**Figure 5:**
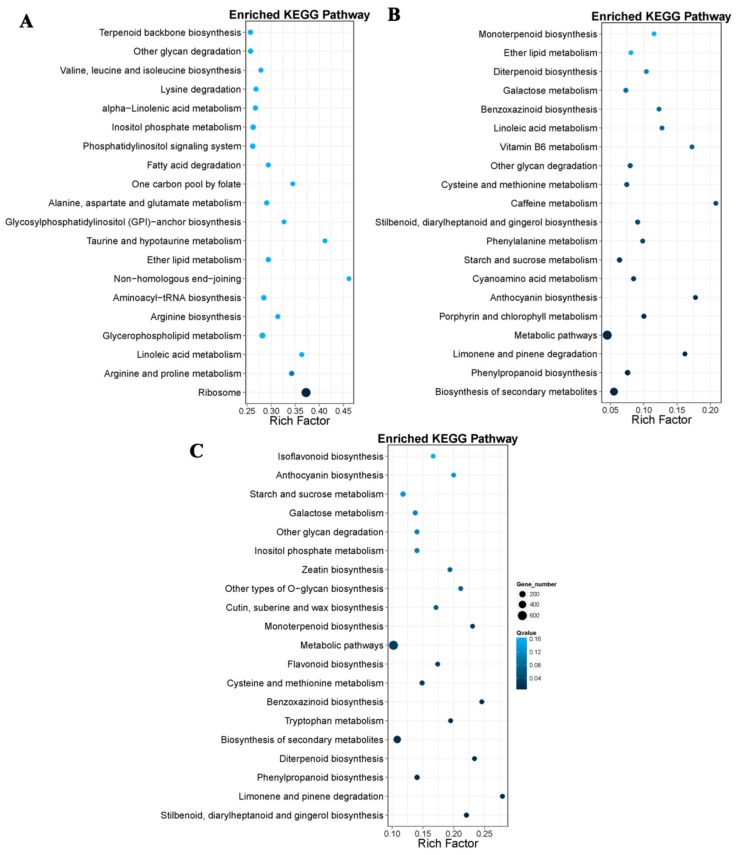
Statistics of pathway enrichment of DEGs in CML530 vs. QCL8011_2 at 18 DAP (**A**), 28 DAP (**B**), and 38 DAP (**C**). X axis means Rich Factor, and Y axis represents second KEGG pathway terms. Rich Factor is the ratio of the differentially expressed gene numbers annotated in this pathway term to all the gene numbers annotated in this pathway term. Greater Rich Factor means greater intensiveness. Q value is corrected *p* value ranging from 0 to 1, and less Q value means greater intensiveness.

For further functional characterization of DEGs, we analyzed the pathway enrichment in DEGs based on the KEGG database. At 18 DAP, 28 DAP, and 38 DAP, 3,547, 608, and 1,406 DEGs were annotated into 19 pathways, respectively, and most genes were annotated as related to the metabolism. Among the DEGs related to the metabolic pathways, 297 DEGs were related to the amino acid metabolism at 18 DAP, and 46 DEGs were involved in lysine metabolism, 23 DEGs were involved in tryptophan metabolism (Figure 5A). A total of 69 DEGs were related to amino acid metabolism at 28 DAP, 8 DEGs involved in lysine metabolism, and 10 DEGs involved in tryptophan metabolism (Figure 5B). Of the 135 DEGs related to amino acid metabolism at 38 DAP, 23 DEGs were involved in lysine metabolism and 24 DEGs were involved in tryptophan metabolism (Figure 5C).

### 3.6. Differentially Expressed Genes Involved in Lysine Metabolism

**Figure 6:**
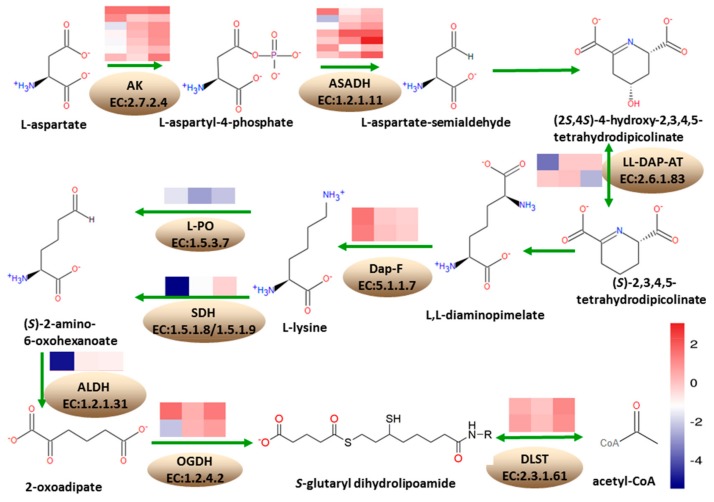
Expression of DEGs and their functions in lysine metabolism. Depths of color in the red and blue rectangles indicate higher and lower Z-score RNA expression lever. The columns on the heat map represent the stage (18 DAP, 28 DAP, and 38 DAP). The rows on the heat map represent the genes. Identified enzymes include: AK—aspartate kinase; ASDH—aspartate-semialdehyde dehydrogenase; LL-DAP-ATL, L-diaminopimelate aminotransferase; Dap-F—diaminopimelate epimerase; L-PO—L-pipecolate oxidase; SDH—saccharopine dehydrogenase; ALDH—aldehyde dehydrogenase; OGDH—2-oxoglutarate dehydrogenase; DLST—dihydrolipoamide succinyltransferase.

According to the KEGG annotation results, at 18 DAP, 13 DEGs were annotated into the lysine biosynthesis pathway, with five being upregulated and eight downregulated. A total of 33 DEGs were annotated into the lysine degradation pathway, where four were upregulated while 29 were downregulated. At 28 DAP, three DEGs were annotated into lysine biosynthesis pathway, where two were upregulated and one was downregulated. Five DEGs were annotated into lysine degradation pathways, where two were upregulated and three were downregulated. At 38 DAP, five DEGs were annotated into lysine biosynthesis pathway, where four were upregulated and one was downregulated. A total of 18 genes were annotated into lysine degradation, where 14 were upregulated while four were downregulated (Figure 6).

Under the lysine biosynthesis metabolism, seven DEGs encode aspartate kinase (AK, EC: 2.7.2.4), involved in the conversion of L-aspartate to L-aspartyl-4-phosphate, in the first step of lysine biosynthesis pathway; of these, *Zm00001d050134*, *Zm00001d021142*, *Zm00001d050133*, and *Zm00001d005151,* were downregulated at 18 DAP. Furthermore, *Zm00001d042471* and *Zm00001d023278* were upregulated while *Zm00001d005535* gene was upregulated at 38 DAP. A total of seven DEGs encoded the aspartate-semialdehyde dehydrogenase (ASADH, EC: 1.2.1.11), involved in the conversion of L-aspartyl-4-phosphate to L-asparte-4-semialdehyde in the lysine biosynthesis pathway, and *Zm00001d042275* was upregulated at 18 DAP, while *Zm00001d033836* and *Zm00001d052231* were downregulated. At 28 DAP, *Zm00001d012847* was downregulated, while *Zm00001d034765* was upregulated. *Zm00001d009968* and *Zm00001d033837* were upregulated at 38 DAP. *Zm00001d008615* and *Zm00001d028165*, encoding L,L-diaminopimelate aminotransferase (L,L-DAP-AT, EC: 2.6.1.83) involved in the conversion of (2*S*,4*S*)-4-hydroxy-2,3,4,5-tetrahydrodipicolinate to (*S*)-2,3,4,5-tetrahydrodipicolinate, were downregulated at 28 DAP and 38 DAP, respectively. *Zm00001d030677* and *Zm00001d041575*, encoding diaminopimelate epimerase (Dap-F, EC: 5.1.1.7) involved in the conversion of L,L-2,6-diaminopimelate to *meso*-diaminopimelate, were upregulated at 18 DAP.

Under the lysine degradation metabolism, *Zm00001d052079*, encoding saccharopine dehydrogenase (SDH, EC: 1.5.1.8/1.5.1.9) involved in the conversion of lysine to (*S*)-2-aminoadipate-6-semialdehyde in the lysine degradation pathway, was downregulated at 18 DAP and 28 DAP, while *Zm00001d020984* encoding L-pipecolate oxidase (L-PO, EC: 1.5.3.7) was downregulated at 18 DAP, 28 DAP, and 38 DAP. *Zm00001d050495* encoding aldehyde dehydrogenase family seven member A1 (ALDH, EC: 1.2.1.31) in the conversion of 2-aminoadipate-6-semialdehyde to L-2-aminoadipate was downregulated at 18 DAP. *Zm00001d023552* encoding 2-oxoglutarate dehydrogenase (OGDH, EC: 1.2.4.2) in the conversion of 2-oxoadipate to *S*-glutaryl-dihydrolipoamide was upregulated at 18 DAP and 38 DAP, while *Zm00001d021889* was downregulated at 18 DAP. *Zm00001d003923* and *Zm00001d025258* encoding dihydrolipoamide succinyltransferase (DLST, EC: 2.3.1.61) in the conversion of *S*-glutaryl-dihydrolipoamide to glutaryl-CoA were upregulated.

### 3.7. Differentially Expressed Genes Involved in the Tryptophan Metabolism

**Figure 7:**
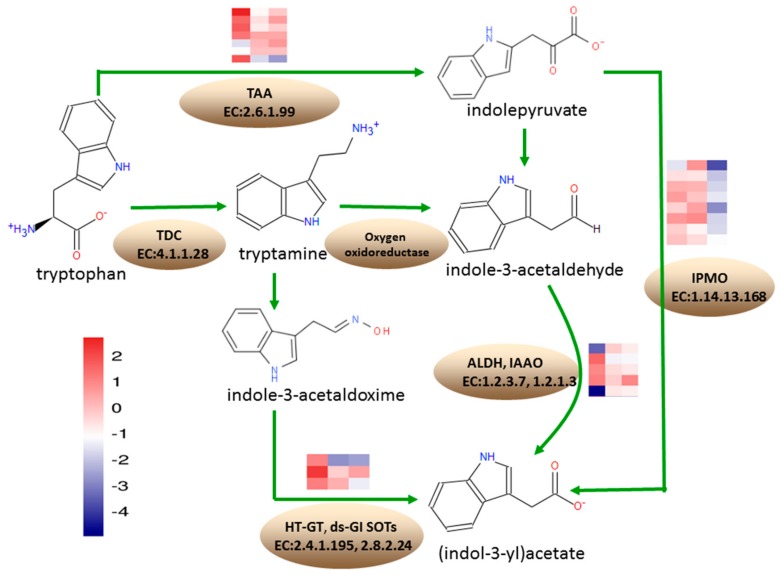
Expression of DEGs and their functions in tryptophan metabolism. Depths of color in the red and blue rectangles indicate higher and lower Z-score RNA expression lever. The columns on the heat map represent the stage (18 DAP, 28 DAP, and 38 DAP). The rows on the heat map represent the genes. Identified enzymes include: TAA—L-tryptophan-pyruvate aminotransferase; IPMO—indole-3-pyruvate monooxygenase; ALDH—aldehyde dehydrogenase; IAAO—indole-3-acetaldehyde oxidase; HT-GT—*N*-hydroxythioamide *S*-beta-glucosyltransferase; ds-GI SOTs—aromatic desulfoglucosinolate sulfotransferase.

According to the KEGG annotation results, at 18 DAP, 23 DEGs were annotated into the tryptophan metabolism pathway, where 13 were upregulated and 10 DEGs were downregulated. At 28 DAP, 10 DEGs were annotated into the tryptophan metabolism pathway, which were all downregulated. At 38 DAP, 24 DEGs were annotated into tryptophan metabolism pathway, where one DEG was upregulated and 23 DEGs were downregulated (Figure 7).

Under tryptophan metabolism, eight DEGs encoding L-tryptophan-pyruvate aminotransferase (TAA, EC: 2.6.1.99), involved in the conversion of tryptophan to indolepyruvate, *Zm00001d037674*, *Zm00001d043650*, *Zm00001d037498*, and *Zm00001d008700*, were upregulated at 18 DAP, while *Zm00001d012728* and *Zm00001d008708* were downregulated. *Zm00001d011562* and *Zm00001d037674* were downregulated at 28 DAP. *Zm00001d043651* was downregulated at 38 DAP. A total of four DEGs encoding aldehyde dehydrogenase (ALDH, EC: 1.2.1.3), involved in the conversion of indole-3-acetaldehyde to indoleacetate, *Zm00001d023580* and *Zm00001d025958*, were upregulated at 18 DAP, while *Zm00001d050495* was downregulated. *Zm00001d004731* was upregulated at 18 DAP but downregulated at 28 DAP and 38 DAP. *Zm00001d034388*, encoding indole-3-acetaldehyde oxidase (IAAO, EC: 1.2.3.7) involved in the conversion of indole-3-acetaldehyde to indoleacetate, was downregulated at 18 DAP. The 8 DEGs encoding indole-3-pyruvate monooxygenase (IPMO, EC: 1.14.13.168), involved in the conversion of indolepyruvate to indoleacetate, were downregulated at 38 DAP. *Zm00001d043165* encoding *N*-hydroxythioamide *S*-beta-glucosyltransferase (HT-GT, EC: 2.4.1.195), involved in the conversion of indole-3-thiohydroximate to indolylmethyldesulfo-glucosinolate, was upregulated, while *Zm00001d00424* was downregulated, at 28 DAP and 38 DAP. *Zm00001d039669* encoding aromatic desulfoglucosinolate sulfotransferase (ds-GI SOTs, EC: 2.8.2.24), involved in the conversion of indolylmethyldesulfo-glucosinolate to glucobrassicin, was downregulated at 38 DAP. 

### 3.8. qRT-PCR Validation

**Figure 8:**
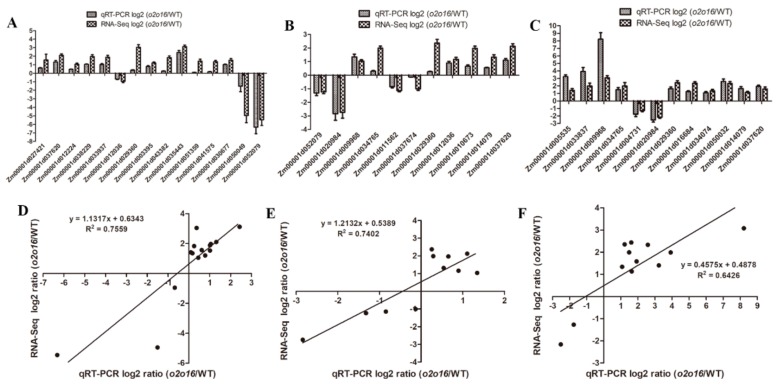
Validation of expression differences identified by RNA-Seq through qRT-PCR of DEGs. (**A**) Histogram of the 15 DEGs in 18 DAP wild-type and *o2o2o16o16* endosperm. (**B**) Histogram of the 11 DEGs in 28 DAP wild-type and *o2o2o16o16* endosperm. (**C**) Histogram of the 12 DEGs in 38 DAP wild-type and *o2o2o16o16* endosperm. (**D**) Scatter plots of the 15 DEGs in 18 DAP wild-type and *o2o2o16o16* endosperm (R^2^ = 0.7559). (**E**) Scatter plots of the 11 DEGs in 28 DAP wild-type and *o2o2o16o16* endosperm (R^2^ = 0.7402). (**F**) Scatter plots of the 12 DEGs in 38 DAP wild-type and *o2o2o16o16* endosperm (R^2^ = 0.6426).

The DEGs identified by RNA-Seq were further validated by qRT-PCR. A total of 28 DEGs were selected for qRT-PCR, which were involved in lysine and tryptophan metabolism. The results showed that the expression patterns of these 28 DEGs were similar to those measured by RNA-Seq (Figure 8A–C). The coefficient of determination, R^2^, between the relative expression levels of qRT-PCR and RNA-Seq, was 0.7559, 0.7402, and 0.6426 at 18 DAP, 28 DAP, and 38 DAP, respectively (Figure 8D–F), indicating the reliability of the RNA-Seq data.

## 4. Discussion

In the present study, we introgressed the *o2* and *o16* alleles into wild-type maize inbred line CML530, using the MABS technique, and acquired the double recessive gene mutant line, QCL8011_2 (*o2o2o16o16*). The lysine content of this line was increased by 56.08%, compared with wild-type line, CML530. By SNP chip scanning, the genetic background recovery rate of QCL8011_2 was 92.62%, which was higher than the theoretical background recovery rate of 87.50%. RNA-Seq analysis showed that, 46, 8, and 23 DEGs were involved in lysine metabolism at 18 DAP, 28 DAP, and 38 DAP, respectively, while 23, 10, and 25 DEGs were mainly involved in the tryptophan metabolism. These could help uncover the transcriptional regulation mechanism in the increase in lysine and tryptophan content, through the introgression of *o2* and *o16* alleles into the wild-type maize.

Hartings et al. determined that Asp, Glu, Gly, Val, Met, Ile, Leu, Phe, Lys, His, and Arg were increased, to varying degrees, in double mutant *o2o2o7o7* seeds, compared with the recurrent parent A69Y [27]. In our study, we found that the contents of Asp, Gly, Phe, Lys, His, and Arg in *o2o2o16o16* were all increased to varying extents, which was consistent with the results tested by Hartings et al. However, the content of Glu, Val, Met, Ile, Leu, and Phe decreased, which was different from the results of Hartings et al. This difference may be related to the introgression of the *o16* allele and the different genetic background (A69Y vs CML530).

Hartings et al. [27] reported that the *o2o7* endosperm showed a reduced transcription level of the 19 and 22 kDa α-zein, and the 10, 27, and 50 kDa γ-zein. Li et al. [37] discovered that O2 could directly bind to the promoters of the known targets of 22 kDa α-zein, 19 kDa α-zein, and 14 kDa β-zein genes. Zhan et al. [38] discovered that O2 directly regulated 23 zein genes, which were, 13 genes of 19 kDa α-zein, 8 genes of 22 kDa α-zein, and one gene, each, of 15 kDa β-zein, 18 kDa δ-zein, 27 kDa γ-zein, and 50 kDa γ-zein. In this study, 16, 15, and 14 genes encoding α-zein were detected at 18 DAP, 28 DAP, and 38 DAP, respectively, and were downregulated in the *o2o2o16o16* mutant as compared to the wild-type (Table S6, Figure S3). Fourteen α-zein genes were detected at all of 18 DAP, 28 DAP, and 38 DAP, of them seven α-zein genes were detected by Zhan et al., and the *GRMZM2G044152* (*Zm00001d048813*) gene was also detected by Li et al. These α-zein genes were downregulated in the *o2o2o16o16* mutant, resulting in a decrease of the zein synthesis and increase in the lysine content. Aspartate kinase (AK, EC: 2.7.2.4) was committed in the first step of the Asp-derived amino acid pathway, in microorganisms and plants, leading to the biosynthesis of essential amino acids, such as lysine, threonine, methionine, and isoleucine [39,40,41,42]. There were at least two isoforms of this enzyme, one feedback inhibited by lysine and the other feedback inhibited by threonine [43]. The gene encoding the lysine-sensitive aspartate kinase (Ask1) might be regulated by the *o2* mutation in maize [44]. The β-aspartyl phosphate produced in the reaction catalyzed by AK, was then converted to β-aspartyl semialdehyde (ASA), in a reaction catalyzed by the enzyme aspartate semialdehyde dehydrogenase (ASADH, EC 1.2.1.11), in an NADPH-dependent reaction [43]. In this study, aspartate kinase homoserine dehydrogenase 2 (akh2), encoding AK involved in the conversion of L-aspartate to L-aspartyl-4-phosphate, was upregulated at 38 DAP in QCL8011_2. ASADH was the second key enzyme in the aspartic acid metabolic pathways, which functions as a catalyst in the conversion of L-aspartyl-4-phosphate to L-asparte-4-semialdehyde. *Zm00001d009968* and *Zm00001d034765*, encoding ASADH involved in the conversion of L-aspartyl-4-phosphateto L-asparte-4-semialdehyde, were upregulated at 28 DAP and 38 DAP in QCL8011_2. *Zm00001d030677* and *Zm00001d041575*, encoding Dap-F involved in the conversion of L,L-2,6-diaminopimelateto *meso*-diaminopimelate, were upregulated at 18 DAP. These changes might promote lysine synthesis and increase the grain lysine content of the QCL8011_2 lines.

Lysine α-ketoglutarate reductase/saccharopine dehydrogenase (LKR/SDH), which was a bifunctional enzyme involved in lysine catabolism, was also regulated by O2 [45]. The O2-regulated LKR/SDH gene (*GRMZM2G181362*), which was involved in lysine catabolism, was downregulated by 4.57-fold in the *o2* endosperm [37]. Kemper et al. found that the transcription level of LKR/SDH mRNA in maize *o2* mutants was decreased by more than 90% and that the enzyme activity was significantly decreased, which reduced the degradation of lysine [45]. In this study, consistent with the above observations, *lkrsdh1* encoding LKR/SDH, involved in the conversion of lysine to 2-aminoadipate-6-semialdehyde, was downregulated at 18 DAP and 28 DAP in QCL8011_2. Meanwhile, *Zm00001d020984*, encoding LOP, was downregulated at 18 DAP, 28 DAP, and 38 DAP. These changes inhibited lysine degradation and increased the grain lysine content of the QCL8011_2.

Trp was first converted to indole-3-pyruvate (IPA) by the TAA family of aminotransferases and, subsequently, indol-3-acetic acid (IAA) was produced from IPA by the YUCCA (YUC) family of flavin monooxygenases. The two-step conversion of Trp to IAA was the main auxin biosynthesis pathway that played an essential role in many developmental processes [46,47]. When we focused on *Zm00001d011562* and *Zm00001d037674*, which encode the TAA involved in the conversion of tryptophan to indolepyruvate, we found that *Zm00001d011562* was downregulated at 28 DAP and *Zm00001d037674* was downregulated at 38 DAP in QCL8011_2. A total of eight DEGs encoding IPMO, involved in the conversion of indolepyruvate to indoleacetate, were downregulated at 38 DAP. The aldehyde dehydrogenase (ALDH) superfamily is composed of a wide variety of enzymes involved in endogenous and exogenous aldehyde metabolism. ALDH enzymes use either NAD^+^ or NADP^+^ as a cofactor to convert aldehydes to their corresponding carboxylic acids and NADH or NADPH [48]. When we focused on *Zm00001d050495* and *Zm00001d004731*, which encode ALDH in the conversion of indole-3-acetaldehyde to indoleacetate in tryptophan metabolism, we found that *Zm00001d050495* was downregulated at 18 DAP and *Zm00001d004731* was downregulated at 28 DAP and 38 DAP. Aldehyde oxidase (AO; EC: 1.2.3.1) could oxidize indole-3-acetaldehyde into indole-3-acetic acid [49]. *Zm00001d034388* encoding IAAO was downregulated at 18 DAP. These changes inhibited the tryptophan degradation and increased the grain tryptophan content of the QCL8011_2.

In this study, the key genes responsible for the increase in lysine and tryptophan content after introgression of *o2* and *o16* alleles into the wild-type maize were identified at the transcriptional level. Among them, genes encoding AK, ASADH, and Dap-F, in the lysine synthesis pathway, were upregulated at different stages of endosperm development (18 DAP, 28 DAP, and 38 DAP), which promoted the synthesis of lysine. Meanwhile, genes encoding LKR/SDH and L-PO in the lysine degradation pathway were downregulated, which inhibited the degradation of lysine. In addition, genes encoding TAA and YUC in the tryptophan metabolic pathway were downregulated, which inhibited the degradation of tryptophan. These would help to uncover the transcriptional regulation mechanisms involved in the increase in lysine and tryptophan content, through the introgression of *o2* and *o16* alleles into wild-type maize.

## 5. Conclusions

In this study, the *o2* and *o16* alleles were introgressd into the wild-type inbred line CML530 by MABS, and a double recessive mutation QCL8011_2 (*o2o2o16o16*) was acquired. The lysine content of QCL8011_2 was improved, compared with that of its recurrent parent. Kernels from QCL8011_2 and CML530 (18 DAP, 28 DAP, and 38 DAP) were subject to the RNA-Seq. The RNA-Seq analysis revealed that 59 DEGs were involved in lysine metabolism, and 43 DEGs were involved in tryptophan metabolism. Among them, the genes encoding AK, ASADH, and Dap-F in the lysine synthesis pathway were upregulated at different stages of the endosperm development (18 DAP, 28 DAP, and 38 DAP), which promoted the synthesis of lysine. Meanwhile, the genes encoding LKR/SDH and L-PO in the lysine degradation pathway were downregulated, which inhibited the degradation of lysine. In addition, genes encoding TAA and YUC in the tryptophan metabolic pathway were downregulated, which inhibited the degradation of tryptophan. These results could help uncover the molecular mechanism in the increase in lysine and tryptophan content, through the introgression of the *o2* and *o16* alleles into the wild-type maize.

## Supplementary Materials

The following are available online at https://www.mdpi.com/2073-4425/10/4/316/s1, Figure S1: The number of identified genes in each sample, Figure S2: The heatmap of correlation coefficient values across samples, A and B represents two biological replicates. Gradient color barcode at the right top indicates the minimum value in white and the maximum in blue. If one sample is highly similar with another one, the correlation value between them is very close to 1, Figure S3. Venn diagram (A) and scatter plot (B) of the 16 zein genes for CML530 vs. QCL8011_2 at 18 DAP, 28 DAP, and 38 DAP, Table S1: Twenty-eight DEGs validated by quantitative real-time PCR analysis, Table S2: Summary for RNA sequencing data of CML530_18 DAP, CML530_28 DAP, CML530_38 DAP, QCL8011_2_18 DAP, QCL8011_2_28 DAP, and QCL8011_2_38 DAP, Table S3: Alignment statistics of reads aligned to the reference genome B73v4, Table S4: QC20 and QC30 of RNA sequencing data for Tai19, QCL3024, QCL5019, QCL8006_1, and QCL8006_2, Table S5: Fragments per kilobase million (FPKM) of all genes for CML530_18 DAP, CML530_28 DAP, CML530_38 DAP, QCL8011_2_18 DAP, QCL8011_2_28 DAP, and QCL8011_2_38 DAP. Table S6: Downregulation of sixteen distinct zein genes in QCL8011_2, compared to CML530 at 18 DAP, 28 DAP, and 38 DAP.

## References

[1] Tian Q.Z., Li X.H., Li M.S., Jiang W., Zhang S.H. (2004). Molecular markers assisted selection to quality protein maize. J. Maize Sci..

[2] Mertz E.T., Bates L.S., Nelson O.E. (1964). Mutant gene that changes protein composition and increases lysine content of maize endosperm. Science.

[3] Sturaro M., Viotti A. (2001). Methylation of the Opaque2 box in zein genes is parent-dependent and affects O2 DNA binding activity in vitro. Plant Mol. Biol..

[4] Rossi V., Motto M., Pellegrini L. (1997). Analysis of the methylation pattern of the maize Opaque-2 (O2) promoter and in vitro binding studies indicate that the O2 B-Zip protein and other endosperm factors can bind to methylated target sequences. J. Biol. Chem..

[5] Holding D.R., Otegui M., Li B., Meeley R.B., Dam T., Hunter B.G., Jung R., Larkins B.A. (2007). The maize floury1 gene encodes a novel endoplasmic reticulum protein involved in zein protein body formation. Plant Cell.

[6] Coleman C.E., Clore A.M., Ranch J.P., Higgins R., Lopes M.A., Larkins B.A. (1997). Expression of a mutant alpha-zein creates the *floury2* phenotype in transgenic maize. Proc. Natl. Acad. Sci. USA.

[7] Nelson Jr O.E. (1976). The location of *fl3* on chromosome 8. Maize Genet. Coop. News Lett..

[8] Mains E.B. (1949). Heritable characters in maize: Linkage of a factor for shrunken endosperm with the *a*_1_ factor for aleurone color. J. Hered..

[9] Teas H.J., Teas A.N. (1953). Heritable characters in maize: Description and linkage of brittle endosperm-2. J. Hered..

[10] Robertson D.S. (1967). A new opaque gene located on chromosome 7. Maize Genet. Coop. News Lett..

[11] Myers A.M., James M.G., Lin Q., Yi G., Stinard P.S., Hennen-Bierwagen T.A., Becraft P. (2011). Maize *opaque5* encodes monogalactosyldiacylglycerol synthase and specifically affects galactolipids necessary for amyloplast and chloroplast function. Plant Cell.

[12] Ashman R.B. (1968). Gene linkages in translocation T9-10a heterozygotes. Maize Genet. Coop. News Lett..

[13] Ma Y., Nelson O.E. (1975). Amino acid composition and storage proteins in two new high-lysine mutants in maize. Cereal Chem..

[14] McWhirter K.S. (1971). A floury endosperm, high lysine locus on chromosome 10. Maize Genet. Coop. News Lett..

[15] Misra P.S., Jambunathan R., Mertz E.T., Glover D.V., Barbosa H.M., McWhirter K.S. (1972). Endosperm protein synthesis in maize mutants with increased lysine content. Science.

[16] Wang G., Sun X.L., Wang G.F., Wang F., Gao Q., Sun X., Tang Y.P., Chang C., Lai J.S., Zhu L.H. (2011). *Opaque7* encodes an acyl-activating enzyme-like protein that affects storage protein synthesis in maize endosperm. Genetics.

[17] Dannenhoffer J.M., Bostwick D.E., Or E., Larkins B.A. (1995). Opaque-15, a maize mutation with properties of a defective opaque-2 modifier. Proc. Natl. Acad. Sci. USA.

[18] Wang G., Zhang J.S., Wang G.F., Fan X.Y., Sun X., Qin H.L., Xu N., Zhong M.Y., Qiao Z.Y., Tang Y.P. (2014). Proline responding1 plays a critical role in regulating general protein synthesis and the cell cycle in maize. Plant Cell.

[19] Salamini F., Di Fonzo N., Fornasari E., Gentinettta E., Reggiani R., Soave C. (1983). *Mucronate*, *Mc*, a dominant gene of maize which interacts with *opaque-2* to suppress zein synthesis. Theor. Appl. Genet..

[20] Yang W.P., Zheng Y.L., Zheng W.T., Feng R. (2005). Molecular genetic mapping of a high-lysine mutant gene (*opaque-16*) and the double recessive effect with *opaque-2* in maize. Mol. Breed..

[21] Zhang W.L., Yang W.P., Chen Z.W., Wang M.C., Yang L.Q., Cai Y.L. (2010). Molecular marker-assisted selection for *o2* introgression lines with *o16* gene in corn. Acta Agronom. Sin..

[22] Sarika K., Hossain F., Muthusamy V., Zunjare R.U., Baveja A., Goswami R., Thirunavukkarasu N., Jha S.K., Gupta H.S. (2018). *Opaque16*, a high lysine and tryptophan mutant, does not influence the key physico-biochemical characteristics in maize kernel. PLoS ONE.

[23] Sarika K., Hossain F., Muthusamy V., Zunjare R.U., Baveja A., Goswami R., Bhat J.S., Saha S., Gupta H.S. (2018). Marker-assisted pyramiding of *opaque2* and novel *opaque16* genes for further enrichment of lysine and tryptophan in sub-tropical maize. Plant Sci..

[24] Jia H., Nettleton D., Peterson J.M., Vazquez-Carrillo G., Jannink J.L., Scott M.P. (2007). Comparison of transcript profiles in wild-type and *o2* maize endosperm in different genetic backgrounds. Crop Sci..

[25] Zhou Z.Q., Song L.Y., Zhang X.X., Li X.H., Yan N., Xia R.P., Zhu H., Weng J.F., Hao Z.F., Zhang D.G. (2016). Introgression of *opaque2* into waxy maize causes extensive biochemical and proteomic changes in endosperm. PLoS ONE.

[26] Wang W., Niu S., Dai Y., Zhai X., Wang M.C., Ding Y., Yang W.P., Zhao D.G. (2019). Molecular mechanisms underlying increase in lysine content of waxy maize through the introgression of the *opaque2* allele. Int. J. Mol. Sci..

[27] Hartings H., Lauria M., Lazzaroni N., Pirona R., Motto M. (2011). The *Zea mays* mutants *opaque-2* and *opaque-7* disclose extensive changes in endosperm metabolism as revealed by protein, amino acid, and transcriptome-wide analyses. BMC Genom..

[28] Cock P.J.A., Fields C.J., Goto N., Heuer M.L., Rice P.M. (2010). The Sanger FASTQ file format for sequences with quality scores, and the Solexa/Illumina FASTQ variants. Nucleic Acids Res..

[29] Jiao Y., Peluso P., Shi J., Liang T., Stitzer M.C., Wang B., Campbell M.S., Stein J.C., Wei X., Chin C. (2017). Improved maize reference genome with single-molecule technologies. Nature.

[30] Kim D., Langmead B., Salzberg S.L. (2015). HISAT: A fast spliced aligner with low memory requirements. Nat. Methods.

[31] Langmead B., Trapnell C., Pop M., Salzberg S.L. (2009). Ultrafast and memory-efficient alignment of short DNA sequences to the human genome. Genome Biol..

[32] Li B., Dewey C.N. (2011). RSEM: Accurate transcript quantification from RNA-Seq data with or without a reference genome. BMC Bioinform..

[33] Tarazona S., Furio-Tari P., Turra D., Pietro A.D., Nueda M.J., Ferrer A., Conesa A. (2015). Data quality aware analysis of differential expression in RNA-seq with NOISeq R/Bioc package. Nucleic Acids Res..

[34] Abdi H., Salkind M.J. (2007). Bonferroni and Šidák Corrections for Multiple Comparisons. Encyclopedia of Measurement and Statistics.

[35] Kanehisa M., Araki M., Goto S., Hattori M., Hirakawa M., Itoh M., Katayama T., Kawashima S., Okuda S., Tokimatsu T. (2008). KEGG for linking genomes to life and the environment. Nucleic Acids Res..

[36] Liu Y.B., Qin L.J., Han L.Z., Xiang Y., Zhao D.G. (2015). Overexpression of maize *SDD1* (*ZmSDD1*) improves drought resistance in *Zea mays* L. by reducing stomatal density. Plant Cell.

[37] Li C.B., Qiao Z.Y., Qi W.W., Wang Q., Yuan Y., Yang X., Tang Y.P., Mei B., Lu Y.D., Zhao H. (2015). Genome-wide characterization of cis-acting DNA targets reveals the transcriptional regulatory framework of *opaque2* in maize. Plant Cell.

[38] Zhan J., Li G., Ryu C., Ma C., Zhang S., Lioyd A., Hunter B.G., Larkins B.A., Drews G.N., Wang X. (2018). Opaque-2 regulates a complex gene network associated with cell differentiation and storage functions of maize endosperm. Plant Cell.

[39] Miyajima R., Shio I. (1972). Regulation of aspartate family amino acid biosynthesis in *Brevibacterium flavum*. V. Properties of homoserine kinase. J. Biochem..

[40] Liberles J.S., Thorolfsson M., Martinez A. (2005). Allosteric mechanisms in ACT domain containing enzymes involved in amino acid metabolism. Amino Acids.

[41] Rinder J., Casazza A.P., Hoefgen R., Hesse H. (2008). Regulation of aspartate-derived amino acid homeostasis in potato plants (*Solanum tuberosum* L.) by expression of *E. coli* homoserine kinase. Amino Acids.

[42] Lo C.C., Bonner C.A., Xie G., D’Souza M., Jensen R.A. (2009). Cohesion group approach for evolutionary analysis of aspartokinase, an enzyme that feeds a branched network of many biochemical pathways. Microbiol. Mol. Biol. Rev..

[43] Azevedo R.A.D., Lancien M., Lea P.J. (2006). The aspartic acid metabolic pathway, an exciting and essential pathway in plants. Amino Acids.

[44] Brennecke K., Neto A.J.S., Lugli J., Lea P.J., Azevedo R.A. (1996). Aspartate kinase in the maize mutants *ASK1-LT19* and *OPAQUE-2*. Phytochemistry.

[45] Kemper E.L., Neto G.C., Papes F., Moraes K.C.M., Leite A., Arruda P. (1999). The role of *opaque2* in the control of lysine-degrading activities in developing maize endosperm. Plant Cell.

[46] Zhao Y. (2012). Auxin biosynthesis: A simple two-step pathway converts tryptophan to indole-3-acetic acid in plants. Mol. Plant.

[47] Tao Y., Ferrer J.L., Ljung K., Pojer F., Hong F., Long J.A., Li L., Moreno J.E., Bowman M.E., Ivans L.J. (2008). Rapid synthesis of auxin via a new tryptophan-dependent pathway is required for shade avoidance in plants. Cell.

[48] Brocker C., Vasiliou M., Carpenter S., Carpenter C., Zhang Y., Wang X., Kotchoni S.M., Wood A.J., Kirch H.H., Kopečný D. (2013). Aldehyde dehydrogenase (ALDH) superfamily in plants: Gene nomenclature and comparative genomics. Planta.

[49] Koshiba T., Saito E., Ono N., Yamamoto N., Sato M. (1996). Purification and properties of flavin-and molybdenum-containing aldehyde oxidase from coleoptiles of maize. Plant Physiol..

