# Supplementary figures and images for: Transcriptome Dynamics of Double Recessive Mutant, o2o2o16o16, Reveals the Transcriptional Mechanisms in the Increase of Its Lysine and Tryptophan Content in Maize
Source: Genes (Basel). 2019 Apr 23;10(4):316. doi: 10.3390/genes10040316 (PMC6523931; doi:10.3390/genes10040316)

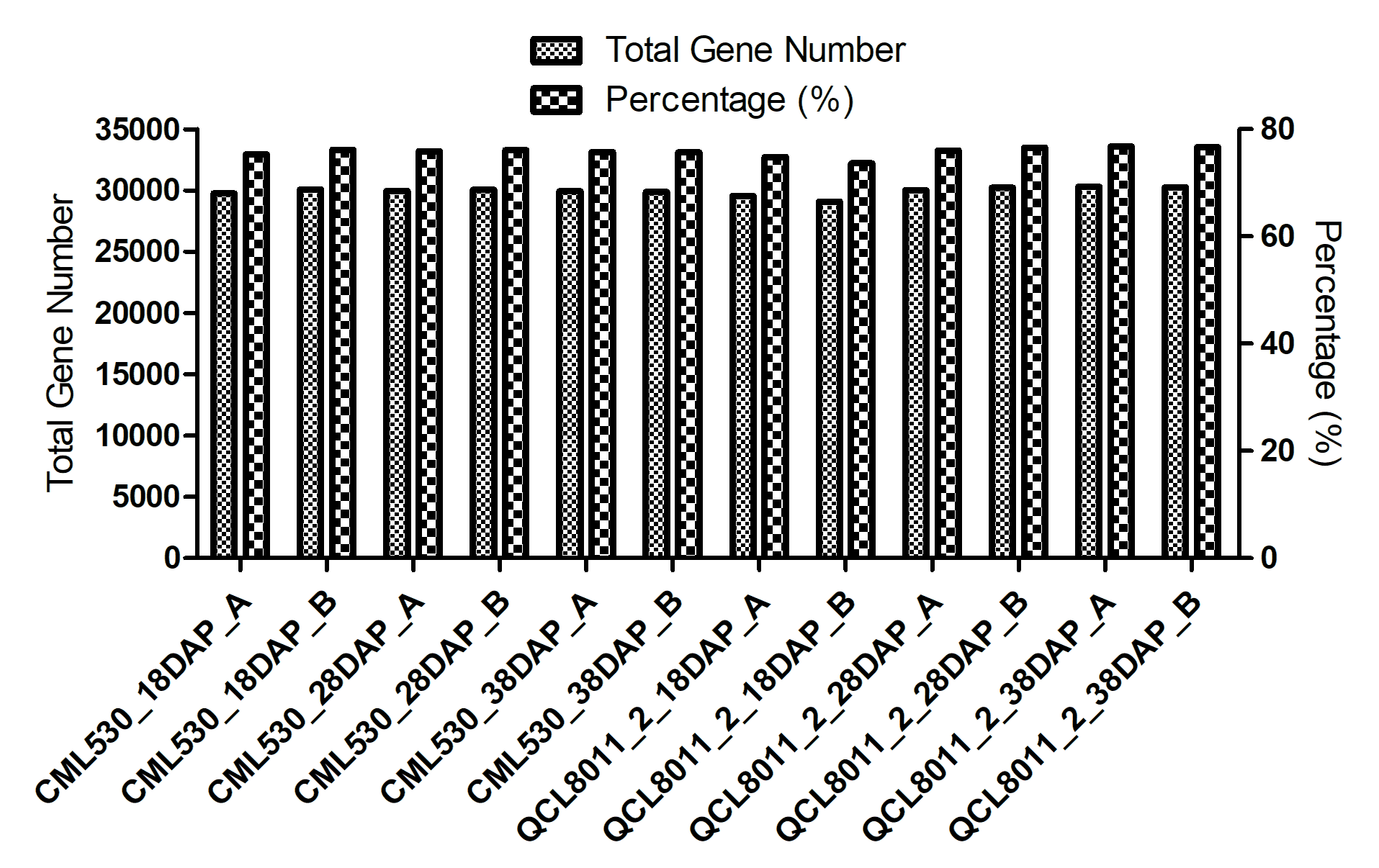

Supplement: Supplementary file 1 [file genes-10-00316-s001.zip › Figure S1-S3/Figure S1. The number of identified genes in each sample.tif]

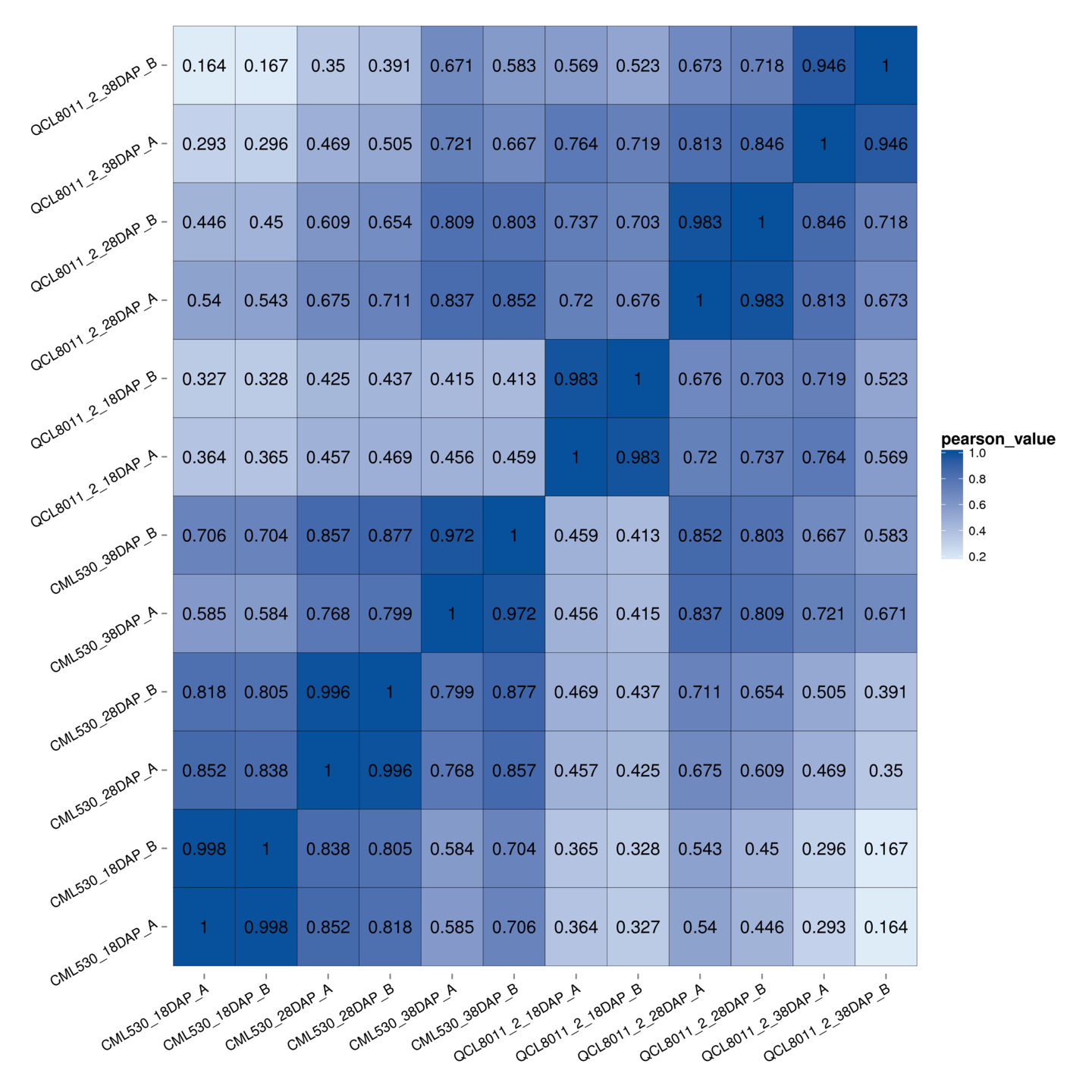

Supplement: Supplementary file 1 [file genes-10-00316-s001.zip › Figure S1-S3/Figure S2. The heatmap of correlation coefficient values acrossing samples..png]

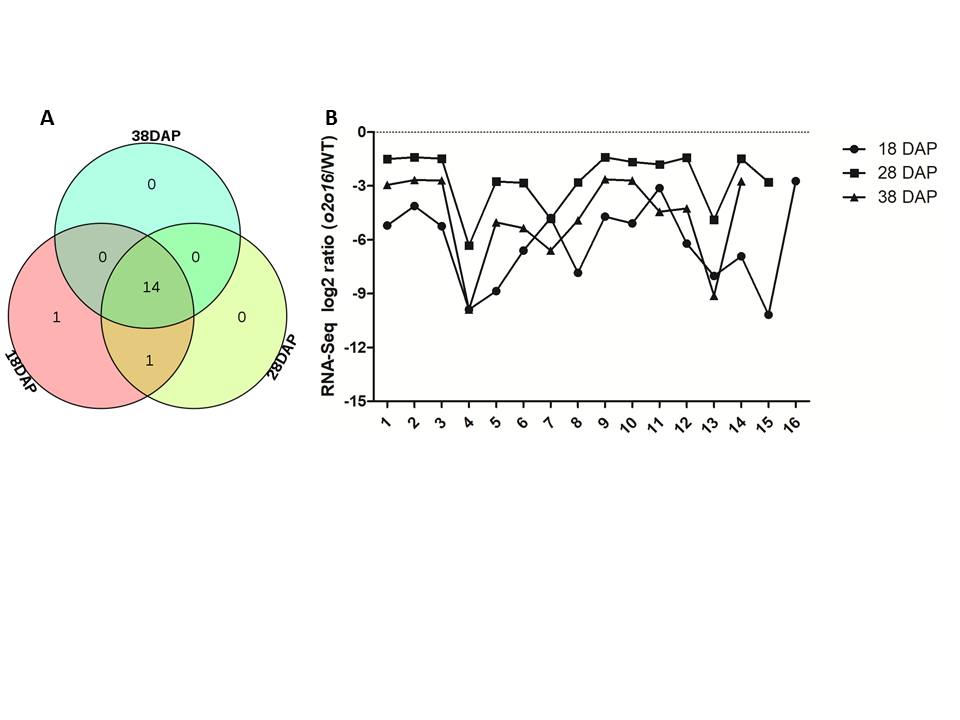

Supplement: Supplementary file 1 [file genes-10-00316-s001.zip › Figure S1-S3/Figure S3.Venn diagram (A) and scatter plot (B) of 16 zein genes for CML530 vs. QCL8011_2 at 18DAP, 28DAP and 38 DAP.tif]
